# Tubulointerstitial Biomarkers for Diabetic Nephropathy

**DOI:** 10.1155/2018/2852398

**Published:** 2018-02-08

**Authors:** Bancha Satirapoj

**Affiliations:** Division of Nephrology, Department of Medicine, Phramongkutklao Hospital and College of Medicine, Bangkok, Thailand

## Abstract

Patients with diabetic nephropathy have a higher risk of mortality, mostly from cardiovascular complications. Standard biomarkers including serum creatinine, estimated glomerular filtration rate, and albuminuria are imprecise, do not directly measure renal tissue injury, and are relatively insensitive to small changes in renal function. Thus, availability of novel biomarkers that are sensitive, specific, and precise as well as able to detect kidney injury and predict clinically significant outcomes would be widely useful in diabetic nephropathy. Novel biomarkers of the processes that induce tubulointerstitial changes may ultimately prove to better predict renal progression and prognosis in type 2 diabetes. Recently, certain biomarkers, which were initially identified in acute kidney injury, also have been reported to confer value in evaluating patients with chronic kidney disease. Biomarkers such as cystatin C, kidney injury molecule-1 (KIM-1), neutrophil gelatinase-associated lipocalin (NGAL), angiotensinogen, periostin, and monocyte chemoattractant protein-1 (MCP-1) reflect tubular injury. In this article, we focused on the potential applications of these biomarkers in diabetic nephropathy.

## 1. Introduction

In 2010, the worldwide adult population with diabetes mellitus was estimated to be about 285 million and by 2030 an increase by 54% to about 439 million is predicted, reflecting a remarkable increase in diabetes nephropathy [1]. Diabetic nephropathy develops along with generalized microvascular disease, most often concomitant with macrovascular disease including cardiovascular, cerebrovascular, and peripheral arterial diseases [2, 3]. Patients with diabetic nephropathy have a higher risk of mortality, mostly from cardiovascular complications, than diabetic patients without nephropathy [4]. According to the Thailand Renal Replacement Therapy (TRRT) Registry Report 2014, the most common cause of end-stage renal disease (ESRD) is diabetes mellitus.

Diabetic nephropathy is a multifactorial disease involving different pathogenic molecular processes and histopathological structure. A combination of renal tubulointerstitial biomarkers that could capture different pathogenic processes of renal injury may provide a more realistic picture of a patient's outcome. More recently, renal tubulointerstitium has been increasingly reported to play an integral role in the pathogenesis of diabetic nephropathy and correlates well with progressive renal function decline [5]. Novel biomarkers of the processes that induce tubulointerstitial changes might ultimately prove to be better predictors of renal progression and prognosis in type 2 diabetes (T2DM).

## 2. Standard Biomarkers of Diabetic Nephropathy

Blood urea nitrogen, serum creatinine, formulas to estimated glomerular filtration rate (GFR), proteinuria, and albuminuria are measures currently used to assess the presence and progress of diabetic nephropathy [2, 3]. However, these measures are imprecise, do not directly measure renal tissue injury, and are relatively insensitive to small changes in renal function. Thus, availability of novel biomarkers that are sensitive, specific, and precise as well as able to detect kidney injury and predict clinically significant outcomes would be widely useful in diabetic nephropathy [6].

Urinary albumin is filtered through the glomerulus and later reabsorbed by tubular cells through the megalin-cubilin pathway. Albuminuria is an important marker to predict the progression of diabetic nephropathy [7] and more severe and persistent albuminuria correlates with more rapid progression of diabetic nephropathy and cardiovascular disease among patients with T2DM. Screening for albuminuria should begin at 5-year diabetes duration among patients with type 1 diabetes and at the time of diagnosis among patients with T2DM based on the clinical practice guidelines for diabetic kidney disease outlined by the Kidney Disease Outcomes Quality Initiative (KDOQI) [8]. The preferred screening test is a urine albumin/creatinine ratio with a first-morning void spot collection. A renal biopsy may be necessary to confirm the clinical diagnosis when atypical features are present. Currently, 20–40% of patients with T2DM show GFR that declines before detecting albuminuria [9]. In addition, some studies have shown about 30–45% of diabetic patients with microalbuminuria progressed to macroalbuminuria in 10 years of follow-up; 30% regressed to normoalbuminuria, and 30–40% remained at microalbuminuria level [9]. For these reasons, investigations of new risk markers to predict progression of diabetic nephropathy are suggested.

## 3. Renal Pathology in Diabetic Nephropathy

Renal pathological changes are present among patients with long-standing diabetes before the onset of microalbuminuria [10]. The characteristic light microscopic features of diabetic nephropathy comprise three major lesions: thickened glomerular basement membranes (GBM) and tubular basement membranes, diffuse mesangial expansion, and hyalinosis of afferent and efferent arterioles. However, hyperglycemia-induced metabolic and hemodynamic stimuli are mediators of kidney injury [11, 12]. These activate inflammatory, prooxidant, ischemic, and fibrotic pathways leading to mesangial matrix accumulation; podocyte effacement and loss; GBM thickening; endothelial dysfunction; tubular atrophy, fibrosis, and dropout; tubulointerstitial inflammation; and renal arteriolar hyalinosis. Therefore, pathology in diabetic nephropathy affects all the renal cellular elements including the glomerular endothelium, mesangial cells, podocytes, and tubular epithelium.

Numerous relationships between tubulointerstitial change and functional outcomes have been reported. Interstitial fibrosis is often proportional to tubular atrophy and a strong predictor of the rate of progression from moderate to severe reduction in GFR [13, 14]. Urinary biomarker data in human beings support the view that tubule injury contributes in a primary way, rather than in a secondary manner, to the development of early diabetic nephropathy [6]. Novel tubular biomarkers related to renal injury in diabetic nephropathy could improve risk stratification and prediction.

## 4. Markers of Tubulointerstitial Injury

Many of the discovered biomarkers for diabetic nephropathy were identified by transcriptomic and proteomic analyses of renal tissues after injury. As such, a bias exists toward identifying markers from the renal tubulointerstitium, reflecting its greater mass compared with the vascular and glomerular compartments. Recently, certain biomarkers, which were initially identified in acute kidney injury (AKI), also have been reported to confer value in evaluating patients with CKD. Biomarkers such as cystatin C, kidney injury molecule-1 (KIM-1), neutrophil gelatinase-associated lipocalin (NGAL), angiotensinogen, periostin, and monocyte chemoattractant protein-1 (MCP-1) reflect tubular injury. In this article, we focused on the potential applications of these biomarkers in diabetic nephropathy as shown in Table 1.

### 4.1. Cystatin-C

Cystatin-C is produced from nucleated cells in the body. It has a molecular weight of 13 kDa, is easily filtered by the glomeruli, and is reabsorbed and catabolized by the proximal tubule. From a prospective observational study, urine cystatin C predicted the progression of T2DM with nephropathy [15]. T2DM with rapid renal progression had significantly increased levels of urine cystatin C when compared with the nonrapid renal progression group [16]. Urine cystatin C was an independent predictor of CKD progression in T2DM.

### 4.2. Neutrophil Gelatinase-Associated Lipocalin (NGAL)

NGAL is a 24 kDa secreted glycoprotein. NGAL is expressed in the renal tubular epithelium, and a rise in urinary concentrations may provide an indication of acute renal injury that can be detected before the rise in serum creatinine concentration [17]. Models of acute kidney injury- (AKI-) to-chronic kidney disease (CKD) transition have implicated its role as a potential biomarker of a chronically injured kidney. In patients with CKD, overexpression of NGAL occurs in renal tubular cells, compared with its expression among non-CKD patients [18], and both serum and urinary NGAL represent a novel, independent renal predictor of the severity of renal disease and CKD progression [19]. In a related study carried out among patients with diabetic nephropathy, elevated urine NGAL level was reported to be associated with the progressive course of the disease leading to ESRD [20]. Similarly, in observational follow-up and our cohort study among patients with T2DM, high urine NGAL levels at baseline correlated with rapid decline of estimated GFR levels and increased serum creatinine [16, 20]. It has been postulated that in the setting of diabetic nephropathy, the rise in NGAL levels occurs independently of decreased GFR and reflects tubular injury and inflammation.

### 4.3. Kidney Injury Molecule-1 (KIM-1)

KIM-1 is a type 1 membrane protein expressed on the apical membrane of proximal tubule cells. Its ectodomain is cleaved and released in the lumen of the tubule and finally appears in urine, which is stable [21]. This biomarker is undetected when the kidneys are normal. Thus, it serves as a specific and sensitive biomarker for proximal tubule damage. In an experimental animal model of tubulointerstitial damage from overload proteinuria, tubular KIM-1 expression was limited to areas with inflammation, fibrosis, and tubular damage [22]. One study also confirmed that KIM-1 was associated with tubulointerstitial inflammation and was overexpressed in the tubules of patients with proteinuric nephropathy, including diabetic nephropathy [23]. From a cross-sectional descriptive study, urine KIM-1 increased in T2DM with normoalbuminuria and mildly increased albuminuria [24]. Serum and urine KIM-1 predicted the rapid decline of GFR [16, 25]. Urinary KIM-1 levels were also significantly higher among patients who progressed from macroalbuminuria to late-stage CKD, but it did not predict progression to end-stage renal disease independently of albuminuria [26]. Urinary KIM-1 excretion could become a noninvasive biomarker of tubulointerstitial injury in diabetic nephropathy. However, Mendelian randomization analysis indicated that the inverse association of increased KIM-1 levels with lower GFR was independent of AER and very likely to represent a causal link [26].

### 4.4. Angiotensinogen

Angiotensinogen is the only known substrate for renin. Changes in angiotensinogen could influence renin angiotensin aldosterone system (RAAS) activity and increase intrarenal RAAS components, parallel to the severity of fibrotic renal damage, and have been demonstrated in chronic progressive nephropathy [27, 28]. Renal angiotensinogen is formed primarily in proximal tubular cells and is secreted in tubular fluid. The activated intrarenal RAS was recently proposed to be involved in the progression of renal injury in multiple models of hypertension and in kidney diseases including diabetic nephropathy, immunoglobulin A (IgA) nephropathy, and radiation nephropathy [29, 30]. Urinary angiotensinogen level also correlates with intrarenal angiotensinogen levels and is independently associated with albuminuria and rapid GFR decline in T2DM [16, 28]. In diabetes with normoalbuminuria, urinary angiotensinogen was higher than in controls and demonstrated good performance in differentiating each stage of T2DM from controls [31]. Our research team proposed that angiotensinogen could serve as a potential urinary biomarker to diagnose diabetic nephropathy. It might be useful as an early biomarker of the activation of the RAAS in diabetic nephropathy.

### 4.5. Periostin

Periostin, osteoblast-specific factor 2, is an extracellular matrix protein firstly expressed in bone, is undetected in other main organs including the kidney [32], and is involved in the fibrosis process and tissue remodeling, kidney development, and tubular dedifferentiation in experimental models. Our study described, for the first time, the renal expression and urinary excretion of periostin in rats after CKD and in the kidneys of mice with diabetes [33]. Increased periostin expression in the glomeruli and tubular epithelium in diabetic renal pathology was observed. It serves as a marker of renal tubular injury and also correlates with worsening renal outcomes including serum creatinine, blood urea nitrogen, and estimated GFR in various chronic progressive kidney injuries [34, 35]. Urinary periostin levels were more significantly elevated among patients of normoalbuminuria, microalbuminuria, and macroalbuminuria compared with levels of healthy controls. Increased urine periostin level was significantly correlated with aging, high albuminuria, and decline of GFR [36]. The current study indicated that increased urine periostin levels could be detected among patients with T2DM before the onset of significant albuminuria and produced an associated renal derangement among patients with established diabetic nephropathy.

### 4.6. Monocyte Chemoattractant Protein-1 (MCP-1)

The infiltration of inflammatory cells such as monocytes and macrophages in diseased kidneys is a hallmark of the progression of diabetic nephropathy [37]. MCP-1 as a member of the CC chemokine family is a major factor influencing macrophage accumulation in both animal and human models of renal damage [38]. MCP-1 is upregulated and expressed in the diabetic glomerular and renal tubular epithelium [39, 40], and a rise in urinary MCP-1 levels correlates with the extent of interstitial inflammatory infiltrate [41, 42]. In contrast, epidermal growth factor (EGF), a peptide growth factor, plays a protective role in progressive renal injury. EGF plays an important role in restoring barrier function in the healing phase of renal injury and is also a critical in vivo renal cell survival factor for developmentally mature kidneys [43, 44]. Urinary levels of EGF and EGF/MCP-1 ratios are inversely correlated with the extent of tubulointerstitial damage and determined renal prognosis in glomerulonephritis [45, 46]. Urinary levels of MCP-1 among patients with overt nephropathy were also more significantly elevated compared with levels among patients with normal albuminuria [47]. Our preliminary data found that urine MCP-1 and urine EGF/MCP-1 ratios were independently associated with renal progression among T2DM patients.

## 5. Conclusion

Novel biomarkers of the processes that induce tubulointerstitial changes may ultimately prove to be better predictors of renal progression and prognosis in T2DM. Our research integrated novel tubular biomarkers simultaneously and compared the performance of each tubular biomarker with standard urine albumin. High levels of urine tubular biomarkers such as urine cystatin C, angiotensinogen, KIM-1, and NGAL presented more rapid decline in renal function among Thai patients with T2DM [16]. This suggests that all novel tubular biomarkers would not be simple surrogate indexes of baseline estimated GFR, but markers on their own, predicting nephropathy progression beyond the information provided by serum creatinine and other conventional risk factors.

## Figures and Tables

**Table 1:** Biomarkers for diabetic nephropathy.

Biomarkers	Source cell	Description
*Standard biomarkers*		
Creatinine	Muscle cells	Glomerular filtration markers [8]
		Factors affecting creatinine generation (i) extremes of muscle mass, (ii) extremes of body size, (iii) diet and nutritional status: high protein diet and creatine supplements, (iv) muscle wasting diseases.

Albuminuria	—	Glomerular damage markers [8]
		20–40% of diabetic patients with renal impairment exhibited normal albuminuria [9]
		No detection in tubulointerstitial injury [9]

*Potential biomarkers*		
Cystatin C	Nucleated cells/proximal tubular cells	Serum cystatin: glomerular filtration markers
		Urine cystatin: tubular markers
		Predicted the renal progression of type 2 diabetes [15, 16]

Neutrophil gelatinase-associated lipocalin (NGAL)	Neutrophils/distal tubular cells	Urine NGAL: distal tubular markers
		Increased in response to tubulointerstitial injury [17, 18]
		Predicted the renal progression of type 2 diabetes [16, 20]

Kidney injury molecule-1 (KIM-1)	Proximal tubular cells	Urine KIM-1: proximal tubular markers
		Increased in response to tubulointerstitial injury [22, 23]
		Predicted the renal progression of type 2 diabetes [16, 24–26]

Angiotensinogen	Proximal tubular cells	Urine angiotensinogen: proximal tubular markers
		Increased in response to renal RAAS activation [27, 29, 30]
		Predicted the renal progression of type 2 diabetes [16, 28, 31]

Periostin	Bone/distal tubular cells	Urine periostin: distal tubular markers
		Increased in response to renal fibrosis and inflammation [33, 34]
		Predicted the renal progression of type 2 diabetes [36]

Monocyte chemoattractant protein-1 (MCP-1)	Macrophages, glomerular and tubular cells	Urine MCP-1: glomerular and tubular markers
		Increased in response to renal inflammation [39–42]
		Predicted the albuminuria and renal progression of type 2 diabetes [47]
